# A Dangerous Lunatic

**Published:** 1888-11-10

**Authors:** 


### A DANGEROUS LUNATIC.

The experiences of physicians and surgeons "within the wards" are not always those of happiness and success. It would not be difficult to give a record of tragical occurrences in hospitals and lunatic asylums which would show daily circumstances of risk and danger of which the general public take little account. On October 1st Dr. Macdowell, visiting physician to the Carlow and Kildare Lunatic Asylum, paid one of his usual visits to the institution. When going round one of the male wards a lunatic of powerful build made a sudden and violent attack upon him. Dr. Macdowell, though surprised for the moment, managed to keep him at bay for a short time, and, watching his opportunity, closed with his assailant, and brought him to the ground. The lunatic bit and struggled as only a madman can, and seizing the doctor by the ear almost succeeded in biting a portion off. The doctor, however, managed to hold him down until assistance arrived sufficient to overpower and place him under restraint. Had the physician been nervous, or weak, or unskilled in the ways of lunatics he might have paid for his humanity with his life. The same patient had previously attempted to murder a nun by throwing a brickbat at her head in the workhouse.

